# Potential impact on using aspirin as the primary prevention of adverse pregnancy outcomes in twins conceived using ART

**DOI:** 10.1038/s41598-024-51543-4

**Published:** 2024-01-26

**Authors:** Dongni Huang, Yao Xie, Pingmei Duan, Jiaxin Wang, Jiacheng Xu, Hongbo Qi, Xin Luo

**Affiliations:** 1 The Department of Obstetrics and Gynecology, Women and Children’s Hospital of Chongqing Medical University, Chongqing, 401147 China; 2 The Department of Obstetrics, The First Affiliated Hospital of Chongqing Medical University, Chongqing, 400016 China; 3 Chongqing Key Laboratory of Maternal and Fetal Medicine, Chongqing Medical University, Chongqing, 400016 China; 4 Maternal and Child Health Hospital of Shapingba District, Chongqing, 401331 China

**Keywords:** Risk factors, Drug safety

## Abstract

With the development of assisted reproductive technology, the number of twin pregnancies is increasing year by year. Given the increased risk of pregnancy complications associated with twin pregnancies, and the fact that these babies are rare and difficult to obtain through assisted reproductive technology, clinicians urgently require finding effective and safe drugs to improve pregnancy outcomes. Low-dose aspirin can not only promote placental blood supply, but also effectively anti-inflammatory. Whether Low-dose aspirin can effectively reduce the risk of pregnancy complications in this special group needs to be clarified. We therefore retrospectively analyzed 665 twin pregnancies from assisted reproduction technology, grouped according to aspirin use, and followed pregnancy outcomes to assess bleeding risk. Low-dose aspirin was found to be effective in preventing preeclampsia without a significant risk of bleeding. However, aspirin does not prevent specific complication in twin pregnancies and seems to have a better preventive effect only when the mother is under 30, which should alarm clinicians should not blindly using aspirin in this particular group.

## Introduction

Assisted reproductive technology (ART) has become an integral part of modern medicine since 1978^1^. However, pregnancies from ART might not have a better perinatal outcome than non-assisted pregnancies^2^. On the other hand, growing utilization of assisted reproductive technology (ART) and advanced maternal age births have resulted in an overall increase in the incidence of twin pregnancies^3^. However, comparing singletons, the complication of twin pregnancies with their increased morbidity and mortality has created significant problems. Including preterm labor (PTL), hypertensive disorders of pregnancy, intrauterine growth restriction and scarred uterus^4^. The rate of preterm labor in twins is > 50%, approximately two to three times greater as compared to singleton pregnancies^5^. The relative risk of preeclampsia also has dramatically increase among twins compared to singletons, 3.50 for dichorionic twins and 2.61 for monochorionic twins, respectively^6^. Rates of caesarean section were significantly higher in twin pregnancies than that in singletons^7^. Moreover, clinicians should also be aware of the likelihood of psychological problems in mothers of multiples and women undergoing assisted reproductive treatment because of the higher expectation of their pregnancy outcomes^8^.

Among singletons, preeclampsia and fetal growth restriction often coexist, with related placental pathologie^9^. Strong evidence suggests that initiation of low-dose aspirin (LDA) prophylaxis prior to 16 weeks gestation reduces the relative risk of developing preeclampsia (PE) or delivering a small for gestational age (SGA) neonate^10^. Thus, aspirin has been suggested preventing preeclampsia, fetal growth restriction or birth of a small-for-gestational-age (SGA) neonate^11^. There was a randomised, double-blind, placebo-controlled trial rolled 11,976 women suggested that the incidence of preterm labor in women took aspirin was 11.6%, lower than that in women took aspirin (13.1, RR 0·89 [95% CI 0.81 to 0.98], p = 0.012)^12^. In addition to these potential benefits, clinicians also need to weigh risks, that is using aspirin during pregnancy might increase the risk of postpartum bleeding^13^. However, few studies have reported the effectiveness of LDA in twin pregnancies, especially in twin pregnancies from ART^14^. Accordingly, it was our aim to assess whether there is a beneficial improvement of pregnancy outcomes after LDA use in twin pregnancies from ART.

## Results

In total, 665 twin pregnancies from ART were recruited in this study. Among them, 155 lost to follow-up were excluded for final analysis. Finally, we obtained complete information from 510 pregnancies (253 in the LDA group and 257 in the control group). The study population included 476 DC and 34 MC according to the type of twins, 500 IVF-ET and 10 IUI according to the mode of conception. The baseline characteristics of the twin pregnancies included in the study are shown in Table 1.

**Table 1:** Maternal baseline clinical characteristics.

Characteristics	Total cases (510)
Year, n (%)	
2016	43 (8.43%)
2017	63 (12.4%)
2018	73 (14.3%)
2019	92 (18.0%)
2020	108 (21.2%)
2021	131 (25.7%)
Age (years), n (%)	31.4 ± 3.83
≤ 35	437 (85.7%)
> 35	73 (14.3%)
BMI (kg/m^2^), mean ± SD	21.9 (21.9 ± 2.94)
Parity, n (%)	
Nulliparous	450 (88.23%)
1–3	59 (11.57)
≥ 4	1 (0.20%)
Previous cesarean delivery, n (%)	
Yes	25 (4.90%)
Occupation, n (%)	
Employed	481 (94.31%)
Unemployed	29 (5.69%)
Type of ART	
IUI	10 (1.96%)
IVF-ET	500 (98.04%)
Type of twin pregnancy	
DCDA	476 (93.3%)
MCDA	34 (6.67%)
Gestational age at delivery (week), n (%)	
< 32	9 (1.76%)
32–34	9 (1.76%)
34–37	52 (10.2%)
≥ 37	440 (86.3%)
Mode of delivery, n (%)	
Vaginal delivery	494 (96.86%)
Cesarean delivery	16(3.14%)
Birth weight (g), mean ± SD	2540 (2540 ± 932)
Apgar score in 5 min, mean ± SD	9.89 (9.89 ± 0.41)
Aspirin use, n (%)	
Yes	253 (49.6%)

### Comparison of prevalence of aspirin use in

In this nearly 6-year study, we found that twin pregnancies from ART are increasing. Aspirin use is also increasingly prevalent in this particular group of pregnant women (*χ*^2^ = 75.513, *p* < 0.05) (Table 2). However, the benefits and risks of aspirin are not clear.

**Table 2:** Prevalence of aspirin use.

	Aspirin use		X^2^	p value
	No	Yes		
Years				
2016	7	2	75.513	< 0.001
2017	6	3		
2018	27	25		
2019	217	223		
2020	239	221		
2021	18	32		

### Comparison of complications in twin pregnancies from ART with different aspirin use or not

Complications were compared between the two groups. The incidence of women overall hypertensive disorders of pregnancy was higher in women taking aspirin than in women taking nothing (*χ*^2^ = 4.593,* p* < 0.05). However, the incidence of preeclampsia was lower in women taking aspirin than in women taking nothing (*χ*^2^ = 4.283, *p* < 0.05). Out of we expected, aspirin use did not contribute much to gestational age at delivery. In addition, the incidence of gestational diabetes (*χ*2 = 0.001, *p* = 0.978), placenta accrete (*χ*^2^ = 0.098, *p* = 0.754) and placental abruption (*χ*^2^ = 1.866, *p* = 0.172) were not different between women who received aspirin or not. However, the rate of cesarean section neonatal was lower in aspirin use group than that of the no use group, and the difference was statistically significant (*χ*^2^ = 4.001, *p* = 0.045). Other maternal outcomes were similar between the two groups (Table 3). The protective role of LDA on specific complication in twins, however, was uncertain (*χ*^2^ = 1.623, *p* = 0.203) (Table 4).

**Table 3:** Gestational disorders by aspirin use during pregnancy.

	Aspirin use		X^2^	p value
	No	Yes		
Gestational age at delivery (week)				
< 32	7	2	3.905	0.272
32–34	6	3		
34–37	27	25		
≥ 37	217	223		
Hypertensive disorders of pregnancy—all forms				
No	239	221	4.593	0.032
Yes	18	32		
Preeclampsia				
No	243	248	4.283	0.038
Yes	14	5		
Gestational diabetes				
No	174	171	0.001	0.978
Yes	83	82		
Premature rupture of membranes				
No	205	215	2.385	0.123
Yes	52	38		
Placenta accrete				
No	212	206	0.098	0.754
Yes	45	47		
Placenta previa				
No	248	246	0.227	0.634
Yes	9	7		
Placental abruption				
No	256	249	1.866	0.172
Yes	1	4		
ICP				
No	223	225	0.558	0.455
Yes	34	28		
Abnormal amniotic fluid volume				
No	246	240	0.209	0.647
Yes	11	13		
Thyroid dysfunction				
No	227	220	0.221	0.638
Yes	30	33		
Mode of delivery				
Cesarean delivery	245	249	4.001	0.045
Vaginal delivery	12	4

**Table 4:** Specific complication in twins by aspirin use during pregnancy.

	Aspirin use		X^2^	p value
	No	Yes		
Gestational typical disorders in twins*				
No	243	232	1.623	0.203
Yes	14	21		

*The number of mothers who experienced at least one of the 7 outcomes including SGA, SIUGR, TRAP, TTTS, Stillbirth in one of the twins; Stillbirth of twins, Abortion.

### Comparison of bleeding risk in twin pregnancies from ART with different aspirin use or not

Notably, Aspirin use did not increase the risk of bleeding during delivery or postpartum. No difference in Postpartum hemorrhage (*χ*^2^ = 0.063, *p* = 0.801) and Postpartum bleeding volume (24 h after birth) (*p* = 0.0754) between the two groups (Supplementary Fig. 1). Also, there was no observed increase in the transfer to Intensive Care Unit (ICU) (*χ*^2^ = 0.32, *p* = 0.572), postpartum blood transfusion (*χ*^2^ = 0.186, *p* = 0.666) and uterine embolism (*χ*^2^ = 0.221, *p* = 0.638) (Table 5).

**Table 5:** Labor and postpartum bleeding-related complications by aspirin use during pregnancy.

	Aspirin use		X^2^	p value
	No	Yes		
Postpartum hemorrhage				
No	223	225	0.063	0.801
Yes	34	28		
Transfusion therapy				
No	246	240	0.186	0.666
Yes	11	13		
Uterine artery embolization				
No	227	220	0.221	0.638
Yes	30	33		
Transfer to ICU				
No	223	225	0.32	0.572
Yes	34	28		

### Comparison of neonatal outcomes in twin pregnancies from ART with different aspirin use or not

There was no statistical significance of the outcome of neonates between the two groups except neonatal gender (*p* = 0.006). Aspirin may affect uterine artery blood flow, however, neither affect neonatal weight gain (*p* = 0.491) nor cause their weight differences (*p* = 0.966). Unfortunately, aspirin use did not reduce their admission to the NICU (*p* = 0.768) (Table 6).

**Table 6:** Birth and birth-related characteristics by aspirin use during pregnancy.

	Aspirin use		p value
	No	Yes	
Neonatal gender			
Like-sex male	60 (23.3%)	79 (31.2%)	0.006
Unlike-sex	144 (56.0%)	106 (41.9%)	
Like-sex female	53 (20.6%)	68 (26.9%)	
Percent difference in birth weight > 20%, n (%)			
No	229 (89.1%)	216 (85.4%)	0.258
Yes	28 (10.9%)	37 (14.6%)	
Mean birth weight (g), mean ± SD	2528 (2528 ± 967)	2552 (2552 ± 898)	0.775
Smaller birth weight (g), mean ± SD	2334 (2334 ± 477)	2361 (2361 ± 398)	0.491
Difference in birth weight* (g), mean ± SD	388 (388 ± 1712)	382 (382 ± 1643)	0.966
Transfer to NICU			
No	187 (72.8%)	188 (74.3%)	0.768
Yes	70 (27.2%)	65 (25.7%)	

* Weight medium (large-small)/large *100% ≥ 20%.

### Comparison of adverse outcomes associated with age in twin pregnancies from ART with different aspirin use or not

**Figure 1:**
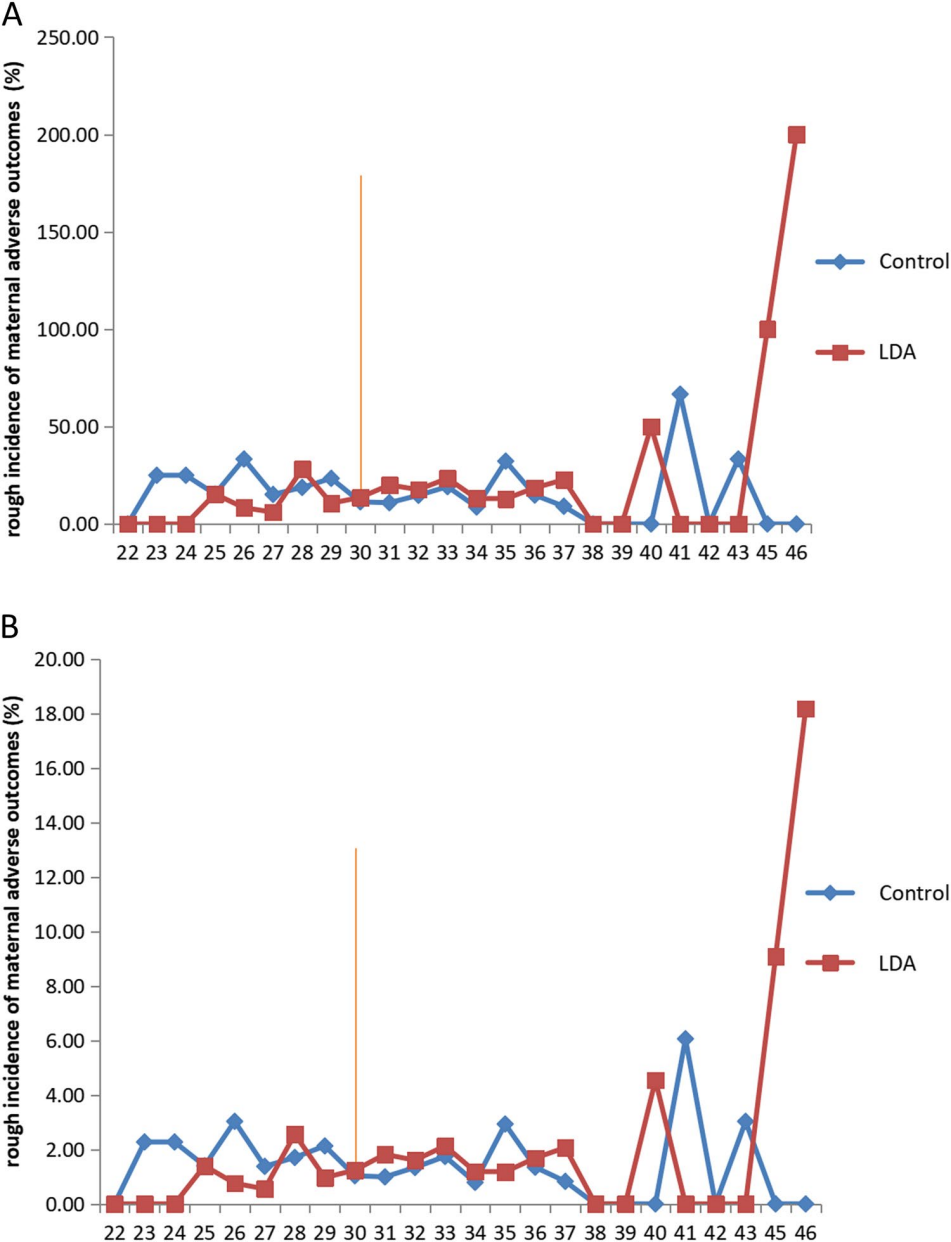
A protective effect of LDA across age stratification: (**A**) the numerator is the number of mothers who experienced at least one of the 8 outcomes, and the denominator is the total number of mothers at the corresponding age. (**B**) Cumulative outcomes, meaning that the numerator is the total frequency of all 8 outcomes, while the denominator is 8 times the number of mothers.

To more fully assess the overall effect of LDA during pregnancy. We further revealed a protective effect of aspirin across age stratification. We chose hypertensive disorder complicating pregnancy, preterm birth, SIUGR, TTTS, TRAP, postpartum hemorrhage, maternal ICU transfer, and intrauterine death as maternal adverse outcomes somewhat arbitrarily. And we found that LDA seemed to be more effective in preventing adverse pregnancy outcomes when the mother was younger than 30 years (Fig. 1).

## Discussion

Twin pregnancies are occurring more frequently with the development of ART, and these women are more likely to develop complications such as gestational hypertension, gestational diabetes, and premature birth^15–17^. There is general consensus on the potential effect of aspirin in early pregnancy for singleton pregnancies to prevent preeclampsia^18–20^, selective fetal restriction^21^, or preterm labor^12^. However, the effect and safety of LDA prophylaxis during pregnancy for twin pregnancies from ART has not been discussed. LDA also seems to have a good preventive effect in Twin pregnancies^22^. Coincidentally, a large proportion of ART pregnant women will take aspirin in the first trimester because they need to increase uterine artery blood flow^23,24^, which leads us to believe empirically that aspirin will bring good prevention effect in this special group of twin pregnancies from ART. As it turns out, the tepid performance of LDA surprised us. Our study found that aspirin does reduce the risk of PE. However, there was no significant protective effect on other outcomes such as preterm labor and fetal weight. In addition, LDA use in the first trimester did not appear to carry a significant risk of prenatal and postnatal bleeding. At the same time, we further found that the overall preventive effect of LDA was affected by age, with the protective effect of aspirin on adverse pregnancy outcomes disappearing at age 30. These findings indicate that LDA can effectively prevent the occurrence of PE, but also has an maternal age limit.

Unlike common anticoagulants such as heparin, LDA the most widely used antipyretic analgesic and anti-inflammatory drug in the world. After hydrolysis in vivo, aspirin is distributed throughout the body as salicylate and can pass through the placenta^25^. As a popular and affordable drug in obstetrics and gynecology, LDA can effectively prevent preeclampsia and fetal growth restriction in singleton pregnancy due to its effect on improving placentation^26^. Aspirin’s role in preventing preterm labor requires further investigation, with some studies showing that LDA can prevent iatrogenic preterm labor caused by preeclampsia^27^ and others showing that aspirin can reduce the risk of recurrent preterm births^28^. But there is little strong evidence that LDA as an anti-inflammatory prolongs gestational weeks and prevents spontaneous preterm labor. Onset of preterm labor remains multifactorial with inflammation and immunological disorders. LDA could downregulate many inflammatory factors^29^, could theoretically lower the incidence of PTL as an immunomodulatory agent. We were fortunate to find that LDA was still effective in preventing PE in twin pregnancies from ART, however LDA was not found to prevent selective fetal restriction or other specific complication in twin pregnancies. Nor did it prolong pregnancy and prevent spontaneous preterm labor. Pregnancy complications in twin pregnancies have more complex mechanisms than in singleton pregnancies, so LDA may not solve all of these problems. Selective fetal restriction does have a similar pathogenesis to hypertensive disorders of pregnancy^14,30^, LDA may prevent preeclampsia by altering placental blood supply, however, in twin idiopathic disorders, such as TRAP and TTTS, most are caused by abnormal placental angiogenesis during early embryogenesis or abnormal development of the heart in one of the fetuses^31,32^. LDA alone may not be able to reverse this problem. Even if ART does not increase major obstetric complications and perinatal risk in twin pregnancies^33^, women who need ART may have underlying problems themselves that increase the risk of adverse pregnancy outcomes. This may also be why LDA does not have the desired overall effect.

Besides, a number of studies have also questioned LDA, which may connect to increased bleeding such as placental abruption and postpartum hemorrhage, leading to concerns that this harmful side effect may outweigh its benefits^34–37^. However, low-dose aspirin in our study did not increase the risk of bleeding, which may be due to our dose selection of 100 mg/day and timely discontinuation in the third trimester. As is known to all, LDA’s potential mechanisms for preventing preeclampsia were to improve blood supply to uterine arteries and platelet aggregation^38^. While, in our study, LDA use still did not increase the incidence of uterine artery embolism, suggesting that the potential risk of LDA is smaller than we expected.

Some previous studies found a frequency of adverse pregnancy outcomes and maternal age^39^. As an independent risk factor, the risk of pregnancy outcome associated with age cannot be reduced by LDA alone. The current definition of advanced maternal age is delivery at age 35 or older^40^. However, in our study, LDA appeared to be more protective against complications of pregnancy under 30. There have also been many studies in recent years devoted to finding age cut-offs at risk for singleton pregnancies. Due to ethnic and regional differences, age cutoffs vary, but 30 years old was associated with the absolute risk of pregnancy^41^. Our results may provide more precise age criteria for LDA use in this particular population of twin pregnancies from ART.

## Highlights

To our knowledge, this is a novel study in the obstetrics field, which revealed the effectiveness and risk of LDA in twin pregnancies from ART. To enable clinical workers to correctly face the advantages and disadvantages of drug use, and effectively improve the pregnancy outcome of specific pregnant women on the premise of ensuring the safety of drug use.

## Conclusion

As LDA became more widely available, we found that LDA did not have the totally same therapeutic effect in twin pregnancies from ART. LDA (100 mg/days) initiated at early gestational age in twin pregnancies from ART could significantly reduce the risk of PE without increasing the risk of serious bleeding-related complications. This study focuses on the advantages and disadvantages of LDA application and provides a multi-dimensional reference for clinical work.

## Materials and methods

### Study population

This study was a tertiary hospital-based retrospective cohort study that included women with twin pregnancies from ART between January 2016 and December 2021 were included in the study (n = 665), and their data from electronic standardized prenatal, delivery, and neonatal records. Informed consent was obtained from all participants. Inclusion criteria were: Twins conceived using ART diagnosed by ultrasound in the first visit (gestational age less than 16 weeks); 18–55 years old. Those who did not start their first antenatal care in our hospital were excluded because of their missing maternal prenatal health records. We also excluded women who had reported use of low-molecular-weight heparin or selective serotonin reuptake inhibitors, since we aim to explore the potential benefits of aspirin on twin pregnancies from ART. We finally followed up 510 women (253 in the LDA group and 257 in the control group). The ethics approval was obtained from the Ethics Committee of the First Affiliated Hospital of Chongqing Medical University. All experiments were performed in accordance with relevant guidelines and regulations. Research involving human research participants has been performed in accordance with the Declaration of Helsinki. Enrollment characteristics are presented in Table 1.

### Exposure

Owing the fact that there were few evidences suggest the safety of aspirin given in twin pregnancy from ART, the obstetricians in this study carried out different policies on aspirin use for twin pregnancies from ART randomly and thus made this observational study feasible. 100 mg/days given in first prenatal visit (10–16 gestational weeks) was set as the LDA group. All data on aspirin use were obtained from prenatal care records.

### Outcome measures

The primary outcomes were the incidence of pregnancy complications which was categorized into (1) common complications during pregnancy, such as gestational age at delivery, hypertensive disorders of pregnancy, preeclampsia (PE), gestational diabetes (GDM), premature rupture of membranes (PROM), placenta accreta placenta, previa placental abruption, intrahepatic cholestasis of pregnancy (ICP), abnormal amniotic fluid, abnormal thyroid function, mode of delivery. And secondary outcomes were specific complication in twins included small for gestational age (SGA), selective intrauterine growth restriction (SIUGR), twin reverse arterial perfusion sequence sign (TRAP), twin-to-twin transfusion syndrome (TTTS), stillbirth and abortion. Clinical safety of aspirin was evaluated by the occurrence of postpartum hemorrhage, transfusion. Uterine artery embolization and transfer to ICU. The assessment of birth and birth-related characteristics included sex ratio, birth weight and transfer to NICU.

### Definition

Hypertensive disorders of pregnancy including pregnancy with chronic hypertension, gestational hypertension, Preeclampsia, Eclampsia or HELLP syndrome were defined by blood pressure ≥ 140/90 mmHg associated with proteinuria (> 300 mg/day or not) after 20 weeks of gestational age. Blood pressure was measured by a mercury sphygmomanometer and urine samples were collected and tested by the clinical laboratory in hospital. When calculating the amount of bleeding, intraoperative blood loss was recorded by the container of suction apparatus, while postoperative bleeding volume was counted by weighting. Cesarean postpartum hemorrhage was defined as postoperative bleeding volume > 1000 ml in 24 h, as for vaginal postpartum hemorrhage, was > 500 ml in 24 h. All other complications and adverse pregnancy outcomes were defined following international obstetrical practice.

### Statistical analysis

SPSS version 22.0 software (SPSS Inc., Chicago, USA) was used for all statistical analyses. We described as mean ± standard deviation if continuous variables were in accordance with a normal distribution and examined them by the T test. The correlation coefficient is calculated by chi-square test and Fisher exact test to illustrate the correlation between classified variables those were presented by percentage. P < 0.05 was considered statistically significant.

### Supplementary Information

Supplementary Figure 1.

## Data Availability

All data generated for this study are included in the article, and further inquiries can be directed to the corresponding author.

## Abbreviations

ART: Assisted reproductive technology
PE: Preeclampsia
PTL: Preterm labor
SGA: Small for gestational age
LDA: Low-dose aspirin
GDM: Gestational diabetes
PROM: Premature rupture of membranes
ICP: Intrahepatic cholestasis of pregnancy
SIUGR: Selective intrauterine growth restriction
TRAP: Twin reverse arterial perfusion sequence sign
TTTS: Twin-to-twin transfusion syndrome
