# Supplementary material for: Potential impact on using aspirin as the primary prevention of adverse pregnancy outcomes in twins conceived using ART
Source: Sci Rep. 2024 Jan 26;14:2223. doi: 10.1038/s41598-024-51543-4 (PMC10817890; doi:10.1038/s41598-024-51543-4)
Supplement: Supplementary file 1 — Supplementary Figure 1. [file 41598_2024_51543_MOESM1_ESM.pdf]

## Potential Impact on Using Aspirin as the Primary Prevention of Adverse Pregnancy Outcomes in Twins Conceived Using ART

**Dongni Huang[a,b,c], Yao Xie[d], Pingmei Duan[b], Jiaxin Wang[b], Jiacheng Xu[a,c,], Hongbo Qi[b,c]& Xin Luo[a,c]**

**[a]The Department of Obstetrics, The First Affiliated Hospital of Chongqing Medical University, Chongqing 400016, China**

**[b]Women and Children's Hospital of Chongqing Medical University, Chongqing 401147, China**

**[c]Chongqing Key Labtory of Maternal and Fetal Medicine, Chongqing Medical University, Chongqing 400016, China**

**[d]Maternal and Child Health Hospital of Shapingba District, Chongqing 401331, China**

**Corresponding authors: Hongbo Qi (E-mail: qihongbocy@gmail.com); Xin Luo (E-mail: 14802315@qq.com)**

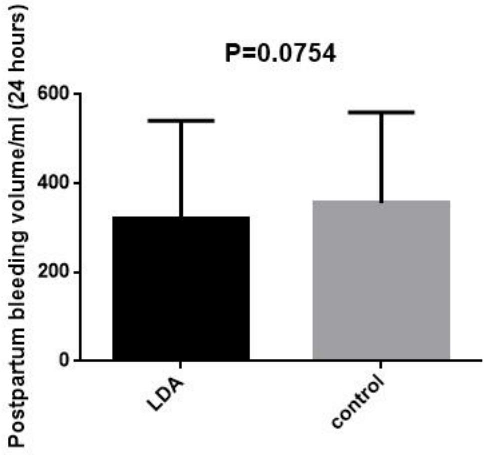


Supplementary Figure 1. *Comparison of Postpartum Hemorrhage Volume in Twin Pregnancies from ART with Different Aspirin use or not*: T test to compare the difference of bleeding volume between the two groups. P>0.05 means no statistical difference
